# Valorization of Heat-Treated Brewers' Spent Grain Through the Identification of Bioactive Phenolics by UPLC-PDA and Evaluation of Their Antioxidant Activities

**DOI:** 10.3389/fnut.2021.634519

**Published:** 2021-04-13

**Authors:** Md. Jiaur Rahman, Lovemore Nkhata Malunga, Michael Eskin, Peter Eck, Sijo Joseph Thandapilly, Usha Thiyam-Hollander

**Affiliations:** ^1^Department of Food and Human Nutritional Sciences, University of Manitoba, Winnipeg, MB, Canada; ^2^Richardson Center for Functional Foods and Nutraceuticals, University of Manitoba, Winnipeg, MB, Canada; ^3^Agriculture and Agri-Food Canada, Richardson Center for Functional Foods and Nutraceuticals, Winnipeg, MB, Canada

**Keywords:** brewers spent grain, heat treated processing, bioactive phenolic, UPLC-PDA, antioxidant properties

## Abstract

Thermal processing not only disrupts cell membranes and cell walls, but also cleaves covalent bonds releasing low molecular phenolic. This study examined the impact of various heat treatments (100, 140, and 160°C) on the composition of phenolic acids and antioxidant activities in extracts obtained from defatted brewers spent grain (BSG) meal. Heating BSG at 160°C resulted in a 2-fold increase in total phenolic content [TPC, 172.98 ± 7.3 mg Gallic acid equivalent (GAE)/100 g defatted meal] and total flavonoid content [TFC, 16.15 ± 2.22 catechin equivalents (CE)/100 g defatted meal] compared to the untreated BSG extracts. The antioxidant activities of treated BSG extracts, determined by radical scavenging and ferric reducing antioxidant power (FRAP) were significantly (*p* < 0.5) higher than the corresponding untreated BSG extracts. Eleven phenolic acids were identified and quantified in BSG extracts by Ultra Performance Liquid Chromatography with Photodiode Array (UPLC-PDA). The amounts varied significantly (*p* < 0.05) depending on the degree of toasting BSG was subjected to. Chlorogenic acid, an ester of caffeic and quinic acid was the predominant phenolic acid present in all fractions. Significant (*p* < 0.05) increases in TPC, TFC, individual phenolic acids and antioxidant activity were observed in BSG extracts exposed to increasing oven temperatures. These results confirm the ability of heat processing to release bioactive phenolic from their bound forms thereby enhancing the phenolic acids and the digestibility of BSG meal in the intestinal tract.

## Introduction

Brewers' spent grain (BGS), a by-product of brewing industry, is produced from barley malt during the production of wort. According to the literature, ~20 kg (WB) of BSG are produced during the production of 100 L beer which represents around 31% of original malted barley weight (1–3). BSG is an inexpensive and underutilized by-product available in large amounts throughout the year (2, 3). It is good source of protein (20% w/w), fiber (70 % w/w) and essential amino acids (2–4). The current application of BGS, however, is limited to its use as an animal feed. Its high nutritional content makes BSG very attractive for enhancing the nutritional value of foods through fortification, as it is both cheap and readily available (3, 4). The potential of BSG as a functional ingredient and a good source of health-promoting bioactive is gradually being recognized. BSG's polyphenols, arabinoxylans, and protein hydrolysate are receiving increasing attention because of their potential health benefits (3, 5).

Phenolic compounds are secondary metabolites present in food grains and are concentrated in the outer layers of the kernels (6). Phenolic compounds are classified into phenolic acids (hydroxycinnamic and hydroxybenzoic acid), flavonoids (flavones, flavonols and anthocyanidins), and tannins based on their molecular structure. Majority of the phenolic compounds in cereal grains are phenolic acids. Hydroxycinnamic and hydroxybenzoic acid can be found free in cell vacuoles or bound to cell wall polysaccharides or lignin. Phenolic acids are also classified based on their solubility in the organic solvents. Soluble phenolic acids include free phenolic acids or those phenolic acids in esterified and etherified to one −2 sugar monomers (7). Conjugated phenolic are generally found in cell vacuoles where they form soluble complex with molecules such as sugars, lipids, proteins, amino acids and other phenolic compound through ester and ether linkages. On the other hand, insoluble phenolic acids exist in the cell walls covalently bound with such cell wall components as polysaccharides (e.g., arabinoxylans), lignin, protein and pectin forming insoluble complexes with each other through ester and ether linkages (8). Both free and conjugate are bioavailable and absorbed in gastrointestinal track. However, insoluble bound phenolic acids cannot be released in the small intestine as humans lack esterase enzymes (9). Thus, efforts to increase the bioavailability of bound phenolic acids through processing is necessary to achieve potential health benefits associated with phenolic acids.

The cumulative phenolic acid concentration of phenolic acids in barley varies with genotype, growing environment, and their interaction. Ferulic acid, caffeic acid, p-coumaric acid, sinapic acid, iso-ferulic acid, p-hydroxybenzoic acid, vannilic acid, and syringic acid have been reported in barley flour (6, 10). Majority of the phenolic acid are found in the pericarp and the aleurone layers barley kernel (11). A number of studies have examined BSG phenolic and their antioxidant activities (10, 12–14). BSG's phenolic acid content is up to 10-fold higher compared to that of barley flour (15–17). The total phenolic acid content in BSG is about 3–5 mg/ g (18) of which <0.5 mg/ g are free (unbound) phenolic acids (19). However, most of the BSG's phenolic Acid Exist in bound form. Thus, this study explored processing technics to increase the amount of free or conjugated phenolic acids in BSG. Thermal treatment of grains has been reported to release bound phenolic (20, 21). Very little data, however, is available on the impact of heat treatment (toasting) on BSG phenolic and their antioxidant activities. This study investigated the effect of oven heat treatments on the extractability of bound phenolic from BSG and analyzed their profiles by both chemical and instrumental methods. Analysis of individual phenolic acids from the BSG extracts was determined by UPLC-PDA and their antioxidant activities using two established methods.

## Materials and Methods

### Chemicals

The organic solvents used in this study were Acetone, hexane, methanol, ethyl acetate, and diethyl ether were obtained from Fisher Scientific Ltd. (Ottawa, ON, Canada). The following chemicals, anhydrous sodium sulfate, sodium hydroxide, sodium chloride, dibasic and monobasic sodium phosphates, sodium nitrite, HC l, AlCl_3_, potassium ferricyanide, ferrous chloride, Folin-Ciocalteu's reagent, ferric chloride, and sodium carbonate were purchased from Fisher Scientific Ltd. (Ottawa, ON, Canada) and Sigma-Aldrich Canada Ltd. (Oakville, ON, Canada). Gallic acid, catechin, Trolox, DPPH radical, and a number of phenolic acid standards were purchased from Sigma-Aldrich Canada Ltd. (Oakville, ON, Canada).

### Sample Collection and Preparation

BSG samples used in the study were kindly supplied by Canadian Malting Barley Technical Center (CMBTC), 303 Main St#1365, Winnipeg, MB R3C 3G7. Prior to heat treatments, BSG was dried at 55^0^C for 24 h in an electric oven (Thelco laboratory oven, Fisher Scientific, Oakville, ON Canada).

### Oven Heat Treatments of BSG

Prior to the extraction of phenolic, BSG samples were pretreated in an oven (Thelco laboratory oven, Fisher Scientific, Oakville, ON Canada) at 100, 140 and 160°C for 30 min. The toasted BSG samples were then ground in a coffee bean grinder (model CBG5 series, Black & Decker, Canada Inc., Brockville, ON, Canada) into a fine powder by passing through a 0.5 mm sieve. The finely ground samples were defatted with hexane in a Soxhlet apparatus at 150°C. The defatted BSG meal was then air-dried followed by the extraction of phenolic.

### Extraction of Phenolic Fraction From Defatted BSG Meals

Phenolic in the defatted BSG meal were extracted by an ultrasound assisted extraction (UAE) technique described by (22) with minor modifications. Both untreated and treated defatted BSG meal (3 g) were mixed with 60 mL of 70% acetone & 30 % water and sonicated for 20 min in an ultrasonic bath (250 HT, 120-volt, 6 Amp, 50-6 Hz, VWR International, and West Chester, PA 19380). Samples were centrifuged for 5 min at 4000 g, 20°C and the supernatants collected. This procedure was repeated one more time. The residue was mixed again with 60 mL of 70% methanol and 30% water following the same procedure as the acetone extraction. Both acetone and methanolic organic supernatants were combined and evaporated to dryness using a rotary evaporator (V-800, 100-240V, 210W, 50-60HZ, Buchi Labortechnik AG, CH-9230 Flawil, and Switzerland) at 40°C. After evaporation, the remaining water phase of the extract was acidified to pH 2 using a 6M HC l solution. The phenolic were extracted from the acidified solution using 6 mL diethyl ether and ethyl acetate (1:1 v/v) with the procedure repeated five times. The combined organic extractant was evaporated under vacuum using a Buchi Rotavapor R-205 (V-800, 100-240V, 210W, 50-60HZ, Buchi Labortechnik AG, CH-9230 Flawil, and Switzerland) at 40°C. Five milliliters of HPLC grade methanol were added to the residue which was stored at −20°C until further analysis.

### Determination of Total Phenolic Content (TPC)

A Folin-Ciocalteu reagent method described by (23, 24) was used to determine total phenolic content (TPC) of BSG extracts. Briefly, 0.5 mL of BSG extract and Folin-Ciocalteu phenol reagent (0.5 mL) were placed in a test tube and mixed using a vortex. After 3 min of incubation, 1 mL of 7.5 % Na_2_CO_3_ was added to neutralize the reaction followed by the addition of 8 mL of distilled water. The tubes were incubated for 35 min at room temperature in the dark. The absorbance of blue color of the supernatant was read at 725 nm using a Diode Array Spectrophotometer (DU 800 Series, Beckman Coulter, Inc., Fullerton, California, USA). The total phenolic content (TPC) in BSG extracts was calculated and expressed as Gallic acid equivalent (GAE) milligram per 100 grams of defatted sample.

### Determination of Total Flavonoid Content (TFC)

The total flavonoid content (TFC) of BSG extracts was determined according to the method by (24). One mL of the BSG extract, 4 mL of d.H_2_0 and 0.3 mL of NaNO_2_ solution (5%) was mixed in the test tubes and allowed to stand for 5 min. Then, 0.3 mL of AlCl_**3**_ solution (10%) was added to each tube. After 1 min, 2 mL of NAOH (1 M), 2.4 mL of distilled water were added and incubated for 15 min in the dark at room temperature. The absorbance was read at 510 nm against a blank prepared devoid of BSG extracts using same amount of methanol. The TFC was calculated from a standard curve for catechin and expressed as mg catechin equivalents (mg CE) per 100 grams of defatted sample.

### Identification and Quantification of Phenolic Acids by UPLC-PDA Analysis

Identification and quantitative analysis of the phenolic acids in treated and non- treated extracts of defatted BSG meal was carried out by an UPLC-PDA method described by (25) with major modifications. The filtrated BSG extracts (0.20 μ m) were analyzed using a reverse-phased Ultra High-Performance Liquid Chromatography [Acquity H class instrument (Waters, Milford, MA)] equipped with a Waters 2,996 Photodiode Array Detector. The sample compartment was maintained at 15°C and the injection volume were set at one microliter. Separation was carried out on a BEH C18 column (Waters Acquity UPLC, 30 mm^*^2.1 mm, 1.8 μm) at 0.7 mL/min flow rate and column temperatures maintained at 30°C. The mobile phase was 0.1% (v/v) acetic acid in water as solvent A and 0.1% (v/v) acetic acid in methanol as solvent B. A gradient elution system operated as follows: 9 - 15% B (0–2 min); 15 - 16.5% B (2 - 2.72 min), 16.5–19% B (2.72–3.17 min), 19–25% B (3.17–3.4 min), 25–26% B (3.4–4.08 min), 26 −28% (4.08–4.31 min), 28–35% B (4.31 − 4.65 min), 35 −40% (4.65 - 5.21 min), 40–48% (5.21–5.44 min); 48–53% B (5.44–6.01 min); 53–70% B (6.01–6.8 min); 70–9% B (6.8–7.37 min); 9–9% B (7.37–7.93 min).

Phenolic acids, such as hydroxybenzoic acids, were monitored at a wavelength of 280 nm while hydroxycinnamic acids were monitored at 320 nm wavelength. The specific identification of each phenolic acid was achieved by comparing the retention times and UV spectral characteristics of the UPLC peak with standard solutions of each type of phenolic acid analyzed under the same conditions. Quantitative determination was performed using an external calibration curve. For this purpose, various phenolic acid standards (e.g., gallic acid, protocatechuic acid, 4-hydroxybenzoic acid, chlorogenic acid, caffeic acid, vanillic acid, syringic acid, ferulic acid, isoferulic acid, *p*-coumaric acid, m-coumaric acid, and sinapic acid) were prepared and diluted to different concentrations in methanol: water (50:50) to obtain the respective calibration curves.

### DPPH Radical Scavenging Assay

DPPH radical scavenging activities of BSG extracts were measured according to the method of (26). Fifty μL of BSG extracts were mixed with 2.95 mL of DPPH radical solution (0.1 mm) in the test tube and vortexed well. A control was prepared identically but devoid of BSG extracts. All tubes were allowed to stand for 10 min at room temperature in the dark. The absorbance of each tube was then read at 516 nm using a Diode Array Spectrophotometer (DU 800 Series, Beckman Coulter, Inc., Fullerton, California, USA). The DPPH radical scavenging effect of BSG extracts were expressed as micromoles Trolox equivalent (TE) per gram of defatted BSG meal.

### Ferric-Reducing Antioxidant Power (FRAP) Assay

The ferric-reducing antioxidant power (FRAP) of BSG extracts was determined as described by (27) with some modifications. 0.5 mL of the BSG extracts, 2.5 mL of a phosphate buffer solution (0.2M, pH 6.6) and 2.5 mL of potassium ferricyanide (1%, w/v) was placed in the test tube and incubated for 20 min at 50°C. Then, 2.5 mL of TCA solution (10%) was added to each tube and the solutions centrifuged for 10 min at 1750 g. A 2.5 mL aliquot of each supernatant was mixed with 2.5 mL of deionized water in separate test tubes. After that, 0.5 mL of FeCl_3_ solution (0.1%; w/v) was added to each mixture and the absorbance read at 700 nm using a UV Spectrophotometer (DU 800 Series, Beckman Coulter, Inc., Fullerton, California, USA). A standard curve was prepared using Trolox. The results were expressed as micromole of Trolox equivalents (TE) per gram of defatted BSG sample.

### Statistical Analysis

All experiments were conducted in triplicate. Data were reported as means ± standard deviation (SD). One-way analysis of variance (ANOVA) and Tukey HSD test identified differences among means using IBM SPSS Statistics version 22 (Armonk, New York, USA).

## Results and Discussion

### Impact of Oven Heat Treatments on the Total Phenolic and Flavonoid Content of BSG

**Figure 1:**
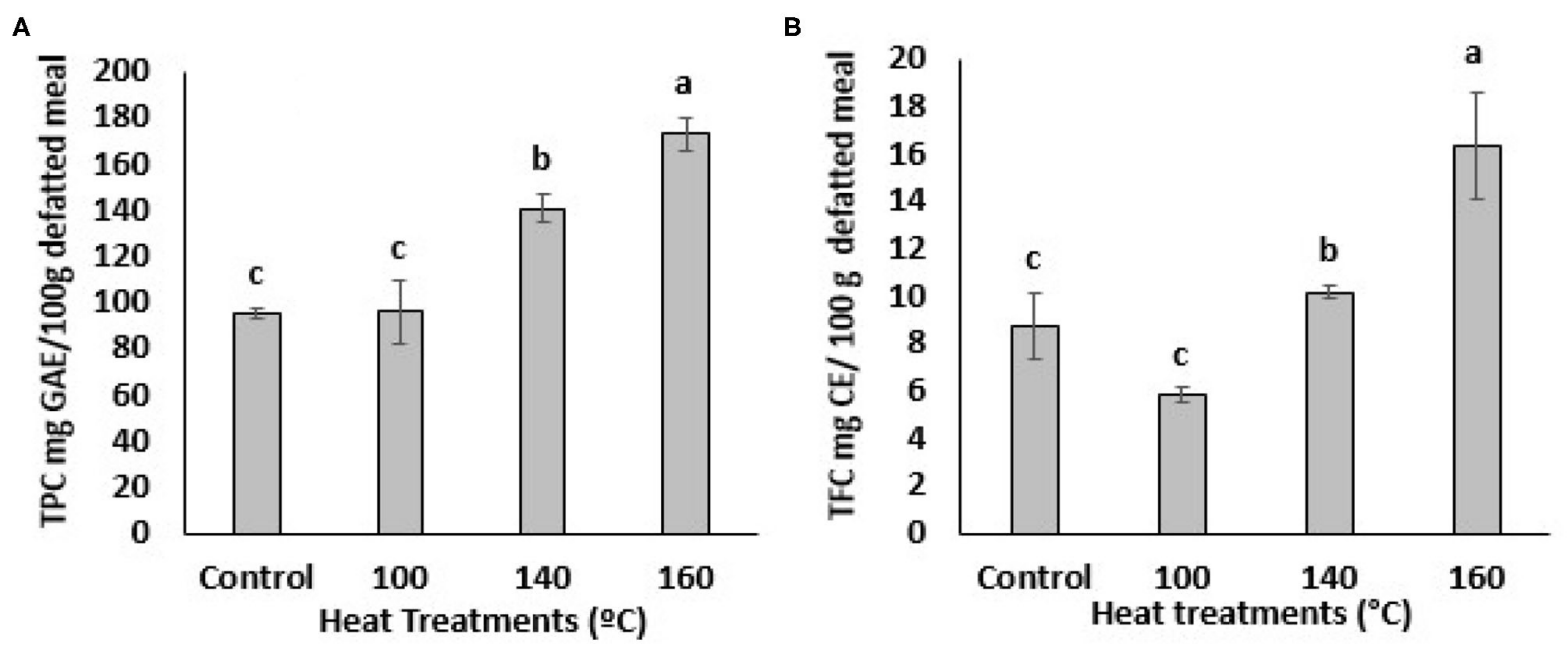
Total Phenolic content (TPC) **(A)** and Total flavonoids content (TFC) **(B)** of BSG. Extracts from defatted meal affected by oven heat treatments. Data represents as mean ± SD (*N* = 3). Mean followed by different letters are significantly different at *p* < 0.05. GAE, gallic acid equivalent; CE, catechin equivalent.

The impact of heat treatments on the TPC of defatted BSG meal is shown in Figure 1A. The TPC value of untreated BSG extract was 96.0 ± 2.27 mg GAE/100 g defatted meal. This value significantly (*p* < *0.05*) increased at temperature above 100°C as determined by Folin Ciocalteu assay (Figure 1A). The BSG extract heated at 160°C showed highest amount of TPC compared to the extracts heated at 100 and 140°C. An almost 2-fold increase in TPC (from 96.0 ± 2.27 to 172.97 ± 7.29 mg GAE/100 g defatted meal) was observed at 160^0^C while BSG treating at 140°C showed 1.5-fold increase in TPC (from 96.0 ± 2.27 to 140.78 ± 5.98 mg GAE/100 g defatted meal) compared to the sample heated at 100°C or the control. This finding was in agreement with previous studies that reported TPC increased significantly in citrus peel, camelina meal, and *Pleurotus eryngii* when heated at the higher temperatures (28–31).

The total flavonoid content (TFC) of the BSG is presented in Figure 1B. Exposure of BSG to 100°C significantly decreased the TFC. Flavonoids are heat sensitive and may undergo thermal degradation (32). Aglycon flavonoids are more susceptible to thermal degradation than the glycosylated ones (33). Contrary, thermal treatment of BSG at 140 °C increased the TFC content compared to the untreated BSG and the difference was statistically significant at *p* < 0.05. TFC of treated BSG extracts also increased significantly (*p* < 0.05) at the higher oven temperatures (160°C). The BSG extract at 160°C was significantly (*p* < 0.05) higher in TFC compared to extracts heated of 100°C, 140°C and the control (Figure 1B). The increase in BSG's TFC at higher temperatures suggest the rate of flavonoid degradation is lower than the rate at which it is being liberated. Thermal degradation products of flavonoids may include phenolic acids (32, 34). Thus, we can stipulate that the thermal degradation of flavonoids may have contributed to the increase in TPC content of BSG. Overall, these results suggest that the highest heat treatment was effective in facilitating the release of the bound phenolic compounds from their esterified and insoluble bound forms in cell vacuoles and walls. However, it was evident that the lower temperature (100°C) was unable to disrupt the cell vacuoles to release phenolic compounds or cleave the covalent bonds between the phenolic compounds and other plant molecules (34). A linear increase was evident for TPC but not TFC when the oven temperature exceeded 100°C. This confirmed the ability of the higher temperatures to release bound phenolic compounds in BSG.

### Impact of Heat Treatment on the Phenolic Acids of BSG

**Figure 2:**
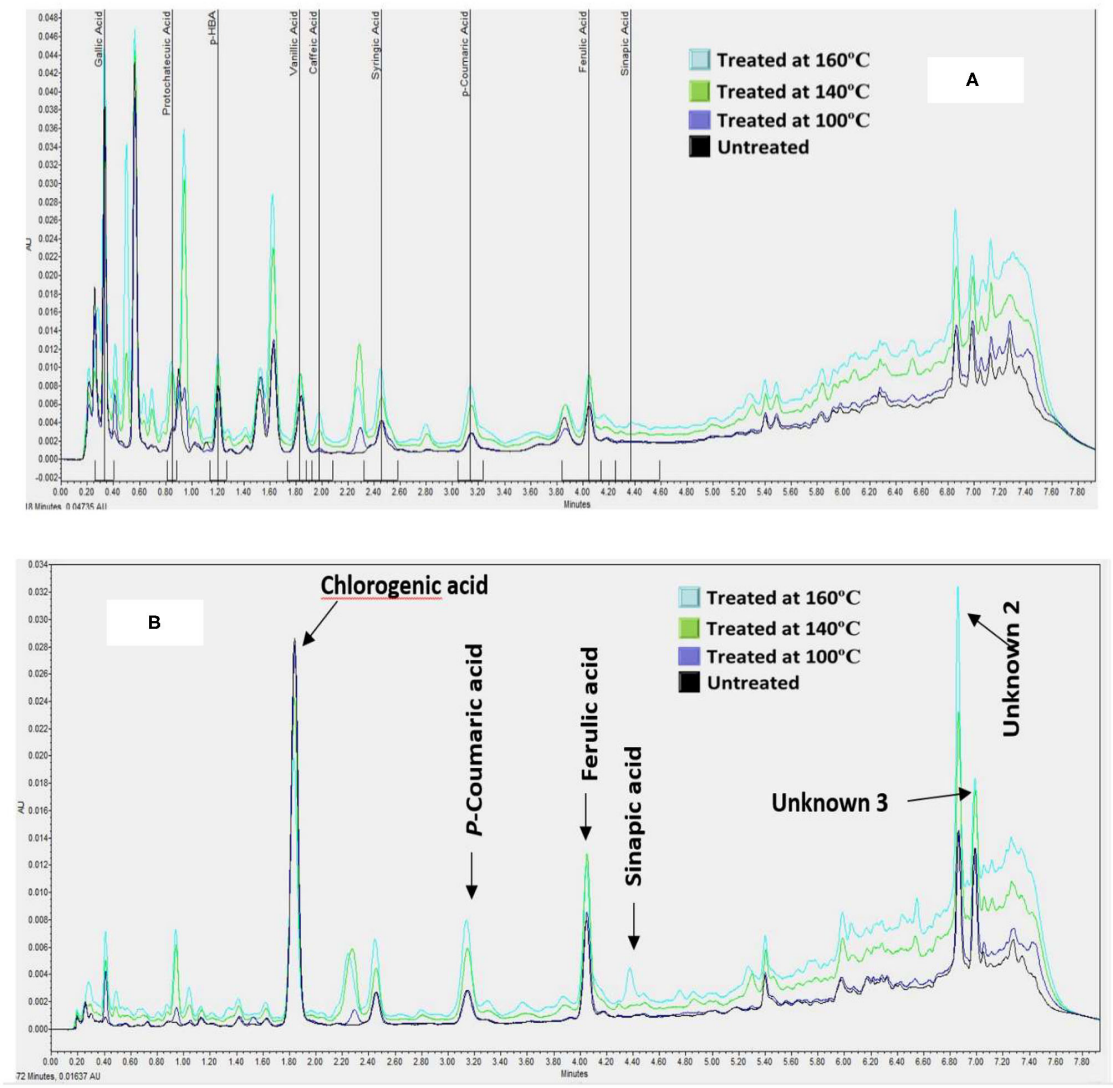
A Chromatogram showing the effect of heat treatments on the composition of hydroxybenzoic acids [at 280 nm, **(A)**] and hydroxycinnamic acids [at 320 nm, **(B)**] of defatted BSG meal analyzed by UPLC-PDA.

The phenolic extracts were separated on a UPLC system to establish the profile and quantify the changes in individual Phenolic compounds in BSG following heat treatments. Twelve (13) phenolic acids were chosen to analyze in this study because these compounds are major phenolic in cereal grains especially in barley and BSG is the outer layer of barley. Among the 12 phenolic acids that were screened for, only 8 were present in the untreated BSG samples namely p-hydroxybenzoic acid, Gallic acid, vanillic acid, protocatechuic acid, chlorogenic acid, syringic acid, p-coumaric acid, and ferulic acid (Figure 2). Similar to TPC, no significant changes were observed in phenolic acid profile when BSG was heated at 100°C. The profile of phenolic acid changed at higher temperature with caffeic acid emerging when BSG was heated at 140°C followed by sinapic acid emerging at 160°C. We also observed three unidentified prominent peaks within the chromatogram that did not correspond to any of the external standards (Figure 2).

The effect of heat treatment on the composition and the concentration of the phenolic acids in the defatted BSG meal extracts is shown in Table 1. The cumulative concentration of the free phenolic acids ranged from 334–458 μg/g of defatted meal. The first two heat treatments (100 and 140°C) did not significantly affect the cumulative concentration of free phenolic acid of BSG. However, the cumulative concentration of phenolic acid increased by 30% when BSG was thermally treated at 160°C. Chlorogenic acid was the predominant free phenolic acid in BSG representing 30% of the total cumulative phenolic acid followed by Gallic acid (17%).

**Table 1:** Impact of heat treatments of defatted BSG meal (DW) on individual phenolic acid content (μg/g sample) analyzed by UPLC-PDA.

	**Oven Heat Treatments**			
**Phenolic acids**	**Untreated/Control**	**Temperature at 100^**°**^C**	**Temperature at 140^**°**^C**	**Temperature at 160^**°**^C**
Gallic acid	57.36 ± 7.81^b^	48.81 ± 7.34^b^	53.90 ± 13.59^b^	82.79 ± 9.30^a^
Unknown 1	76.05 ± 0.92^b^	68.34 ± 4.29^a,b^	72.57 ± 9.81^a,b^	83.62 ± 5.27^a^
Protocatechuic acid	22.39 ± 1.95^b^	18.39 ± 3.75^b^	25.94 ± 4.16^b^	37.41 ± 4.28^a^
4-hydroxybenzoic acid	10.77 ± 0.90^b^	9.95 ± 0.83^b^	13.64 ± 1.70^a,b^	16.59 ± 3.36^a^
Vanillic acid	20.84 ± 0.84^b^	19.03 ± 1.25^b^	23.48 ± 2.36^a,b^	26.59 ± 5.29^a^
Syringic acid	13.72 ± 0.97^c^	15.29 ± 0.75^c^	23.42 ± 2.82^b^	35.65 ± 3.85^a^
Chlorogenic acid	107.18 ± 2.29^a^	102.01 ± 5.36^a^	81.92 ± 10.34^b^	71.28 ± 4.74^b^
Caffeic acid	–	–	3.79 ± 0.49	7.88 ± 0.73
*p*-Coumaric acid	3.71 ± 0.25^c^	3.41 ± 0.22^c^	7.68 ± 0.96^b^	11.42 ± 0.57^a^
Ferulic acid	9.5 ± 0.27^b^	10.2 ± 0.62^b^	15.58 ± 1.13^a^	15.39 ± 1.29^a^
Sinapic acid	–	–	–	3.64 ± 0.025
Unknown 2	23.91 ± 3.17^c^	24.15 ± 2.72^c^	31.73 ± 2.95^b^	41.99 ± 2.04^a^
Unknown 3	17.02 ± 1.92^b^	14.88 ± 0.69^b^	20.68 ± 1.40^a^	23.31 ± 0.98^a^

*Data represents as mean ± SD (N = 3). Mean for the samples followed by the different letters are significantly different at p < 0.05*.

The concentration of the hydroxybenzoic acids such as Gallic, protocatechuic, 4-hydroxybenzoic, vanilic, and syringic acids increased by 44, 68, 60, 30 and 170%, respectively, when BSG was heated to 160°C. Among the hydroxycinnamic acids, the concentrations of ferulic and p-coumaric acid increased significantly by 30 and 200%, respectively. In contrast, of the phenolic acids examined, only chlorogenic acid decreased significantly (*p* < 0.05). These observations suggest the release of bound phenolic acids or the release of phenolic acid trapped in cell vacuole (35). Ferulic acid is mostly bound to arabinoxylans (7) whereas p-coumaric acid is mostly bound to lignin materials. The emergence of caffeic acid may signal the breakdown of chlorogenic acid, an ester of caffeic acid and quinic acid. Thus, the decrease in chlorogenic acid was attributed to its conversion to caffeic acid when BSG was heated at both 140°C and 160°C (Figure 2). The increase in protocatechuic acid is an indication of thermal degradation of flavonoids (32). This confirms the ability of the higher temperatures to cleave the esterified and glycosylated bonds (29). A smaller sinapic acid peak appeared at 160°C at around 3.64 ± 0.025 μg/g of defatted meal indicating that sinapic acid was present in BSG in the bound form. Xu et al. (29) reported that TPC and individual phenolic compounds in the esterified, glycoside and bound fractions from citrus meal decreased with increase in temperature while at the same time there was an increase in the free fraction.

Among the unidentified phenolic compounds (Figure 2), unknown 1 (peak 1) increased from 76.05 ± 0.92 to 83.62 ± 5.27 μg/g of defatted meal, compound 2 (peak 2) from 23.91 ± 3.17 to 41.99 ± 2.04 μg/g of defatted meal; and compound 3 (peak 3) from 17.02 ± 1.92 to 23.31 ± 0.98 μg/g of defatted meal. The future direction which can be taken based on these outcomes is to analyze the phenolic acid profiles of treated and non-treated BSG meal by HPLC-MS/MS.

### Impact of Heat Treatments on the Antioxidant Activities of BSG's Phenolic Extracts

**Figure 3:**
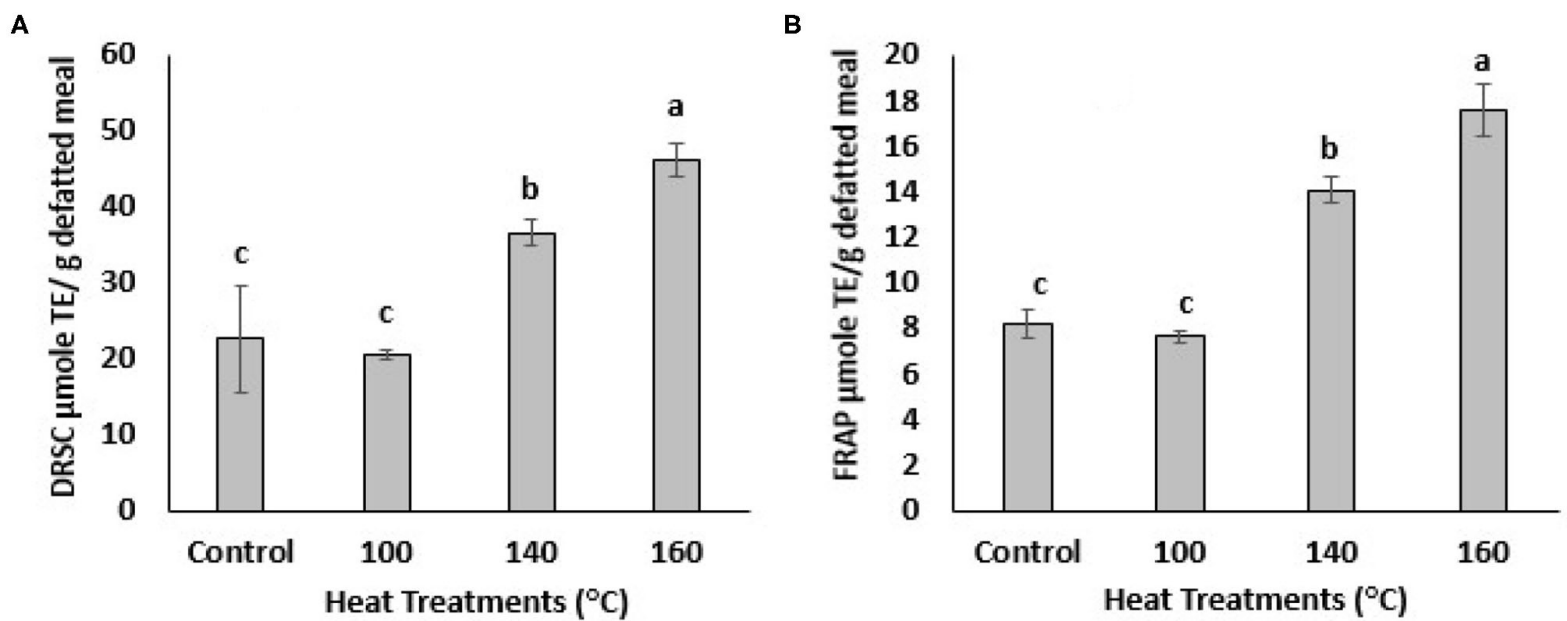
DPPH radical scavenging capacity (DRSC, **A**) and ferric-reducing antioxidant power (FRAP, **B**) of extracts from defatted BSG meal affected by various oven heat treatments. Means followed by the same letters are not significantly different at *p* > 0.05.

The antioxidant activities of BSG phenolic extracts were investigated using 2-well-known assays, DPPH radical scavenging and FRAP assay. DPPH is an artificial free radical generally used to measure antioxidant activities of plant and biological samples of interest. The DPPH radical scavenging capacity (DRSC) of the BSG extracts increased with heating at different temperatures as shown in the Figure 3A. The highest DPPH radical scavenging capacity was observed when treated at 160°C, which also generated the largest amount of TPC and TFC. The DRSC values increased from 22.67 ± 6.93 to 46.26 ± 2.17 μmole/g defatted meal following treatment at 160°C which was 2-folds higher than control. The DRSC value also increased after being heated at 140°C. The ferric-reducing antioxidant power (FRAP) of BSG extracts also increased significantly (*p* < 0.05) with oven heat treatments (Figure 3B). For example, after being heated at 140°C, FRAP values increased significantly (*p* < 0.05) from 8.30 ± 0.49 to 13.83 ± 0.77 μmole/g defatted meal. The highest FRAP value was recorded at 160°C increasing significantly (*p* < 0.05) to 17.27 ± 1.15 μmole/ g defatted meal. Heating BSG at 100°C, however, did not increase DRSC and FRAP value significantly which was consistent with TPC levels which were not significantly different from control at that temperature. These findings were agreement with DRSC and FRAP values published by (28, 29) in which antioxidant activities significantly increased in citrus peel extracts heated at different temperatures. Our results found a significant (*p* < 0.05) increase in TPC in the defatted BSG extracts heated at 140 and 160°C which paralleled the corresponding increase in DPPH radical scavenging capacities and FRAP reducing power. Because of the complexity of the oxidation-antioxidation processes, it is obvious that no single testing method is capable of providing a full picture of the antioxidant profile of a studied sample. A combination of sensitive, rapid and reproducible methods that enable complementary results of hydrogen atom transfer (HAT) and signal electron transfer (SET) based mechanism for antioxidant properties, generally used when an antioxidant activity (AOA) screening is designed. DPPH method used in this study to measure the ability of BSG phenolics (antioxidants) to scavenge free radicals which are primary products in lipid oxidation. FRAP method used in this study to measure the ability of BSG phenolics (antioxidants) to reduce metal ions which are catalyst in lipid oxidation accelerating lipid oxidation. However, to provide a full picture of the antioxidant profile of BSG extracts, assays such as oxygen radical absorbance capacity (ORAC), 2, 2-azinobis (3-ethylbenzothiazoline-6-sulfonic acid) (ABTS), 2, 2-diphenyl-1-picrylhydrazyl (DPPH), reducing power and metal chelation are recommended.

## Conclusion

This study demonstrated a significant (*p* < 0.05) increase in TPC and TFC levels in BSG extracts heated above 100°C with a corresponding increase in their antioxidant activities. Several phenolic acids were identified and quantified by UPLC-PDA in the treated and untreated BSG extracts with chlorogenic acid being the predominant compound present. The higher heat treatments (>100°C) released hydroxybenzoic and hydroxycinnamic acids from their bound forms. Heating the BSG extracts at 160°C resulted in highest levels of TPC and TFC, individual phenolic acids and free radical scavenging activities. These results demonstrated the ability of high oven temperatures to release bioactive phenolic from being bound to the cell wall. Further research is recommended to examine the efficacy of heat treatments in releasing bound phenolic acids in intestinal tract in both *in vitro* and *in vivo* study, respectively as well as analyze their profiles in interest of samples by HPLC-MS/MS.

## Data Availability Statement

The raw data supporting the conclusions of this article will be made available by the authors, without undue reservation.

## Author Contributions

MR performed the experiments, interpreted the results, and drafted the manuscript. LM designed the experiments and conducted the UPLC analysis. ME, UT-H, ST, LM, and PE were involved in proofreading and writing the manuscript. UT-H research program is investigating green technologies for fractionating canola bioactive with a focus on sinapates. All authors contributed to the article and approved the submitted version.

## Conflict of Interest

The authors declare that the research was conducted in the absence of any commercial or financial relationships that could be construed as a potential conflict of interest.
